# Tumour biology, metastatic sites and taxanes sensitivity as determinants of eribulin mesylate efficacy in breast cancer: results from the ERIBEX retrospective, international, multicenter study

**DOI:** 10.1186/s12885-015-1673-3

**Published:** 2015-10-08

**Authors:** Mélodie Dell’Ova, Eléonora De Maio, Séverine Guiu, Lise Roca, Florence Dalenc, Anna Durigova, Frédéric Pinguet, Khedidja Bekhtari, William Jacot, Stéphane Pouderoux

**Affiliations:** 1 Département de Pharmacie Clinique, Institut régional du Cancer de Montpellier (ICM), 208, rue des Apothicaires, 34298 Montpellier Cedex 5, France; 2 Medical Oncology Department, Institut Claudius Regaud, IUCT- Oncopole, Toulouse, France; 3 Medical Oncology Department, Centre Georges-François Leclerc, Dijon, France; 4 Breast Center (CePO), University Hospital CHUV, Rue du Bugnon 46, 1011 Lausanne, Switzerland; 5 Département de Biostatistiques, Institut régional du Cancer de Montpellier (ICM), 208, rue des Apothicaires, 34298 Montpellier Cedex 5, France; 6 Département d’Oncologie Médicale, Institut régional du Cancer de Montpellier (ICM), 208, rue des Apothicaires, 34298 Montpellier Cedex 5, France; 7 Medical Oncology Department, University Hospital of Geneva, Gabrielle-Perret-Gentil 4, 1211 Geneva, Switzerland

**Keywords:** Breast cancer, Eribulin mesylate, Efficacy, Safety

## Abstract

**Background:**

Our retrospective, international study aimed at evaluating the activity and safety of eribulin mesylate (EM) in pretreated metastatic breast cancer (MBC) in a routine clinical setting.

**Methods:**

Patients treated with EM for a locally advanced or MBC between March 2011 and January 2014 were included in the study. Clinical and biological assessment of toxicity was performed at each visit. Tumour response was assessed every 3 cycles of treatment. A database was created to collect clinical, pathological and treatment data.

**Results:**

Two hundred and fifty-eight patients were included in the study. Median age was 59 years old. Tumours were Hormone Receptor (HR)-positive (73.3 %) HER2-positive (10.2 %), and triple negative (TN, 22.5 %). 86.4 % of the patients presented with visceral metastases, mainly in the liver (67.4 %). Median previous metastatic chemotherapies number was 4 [1–9]. Previous treatments included anthracyclines and/or taxanes (100 %) and capecitabine (90.7 %). Median number of EM cycles was 5 [1–19]. The relative dose intensity was 0.917. At the time of analysis (median follow-up of 13.9 months), 42.3 % of the patients were still alive. The objective response rate was 25.2 % (95 %CI: 20–31) with a 36.1 % clinical benefit rate (CBR). Median time to progression (TTP) and overall survival were 3.97 (95 %CI: 3.25–4.3) and 11.2 (95 %CI: 9.3–12.1) months, respectively. One- and 2-year survival rates were 45.5 and 8.5 %, respectively. In multivariate analysis, HER2 positivity (HR = 0.29), the presence of lung metastases (HR = 2.49) and primary taxanes resistance (HR = 2.36) were the only three independent CBR predictive factors, while HR positivity (HR = 0.67), the presence of lung metastases (HR = 1.52) and primary taxanes resistance (HR = 1.50) were the only three TTP independent prognostic factors. Treatment was globally well tolerated. Most common grade 3–4 toxicities were neutropenia (20.9 %), peripheral neuropathy (3.9 %), anaemia (1.6 %), liver dysfunction (0.8 %) and thrombocytopenia (0.4 %). Thirteen patients (5 %) developed febrile neutropenia.

**Conclusion:**

EM is an effective new option in heavily pretreated MBC, with a favourable efficacy/safety ratio in a clinical practice setting. Our results comfort the use of this new molecule and pledge for the evaluation of EM-trastuzumab combination in this setting. Tumour biology, primary taxanes sensitivity and metastatic sites could represent useful predictive and prognostic factors.

## Background

Breast cancer represents the second most common cancer worldwide and the most frequent cancer in women, with an estimated 1.67 million new cases in 2012 (25 % of all cancers) [1, 2]. Anthracyclines and taxanes remain the standard as first-line metastatic breast cancer (MBC) treatment [3]. However, anthracyclines are often given in a neoadjuvant/adjuvant situation and their use in MBC should take into account the risk of cumulative cardiac toxicity. Taxanes are today extensively included in the early breast cancer adjuvant chemotherapy regimens and expose the patients to the risk of peripheral neurotoxicity. Other molecules such as the capecitabine, the liposomal doxorubicin, the pegylated liposomal doxorubicin, the gemcitabine and the vinorelbine, have also been registered, more recently, in the MBC setting [4]. However, no clear standard of care has been validated after first-line treatment failure, and further treatment lines depend mainly on the drugs previously used, their residual toxicities, the performance status of the patient, and the biology and metastatic spreading of the tumour.

Eribulin mesylate (EM, Halaven^®^, E7389) is a new drug indicated for patients with MBC previously treated with an anthracycline and a taxane in the adjuvant or metastatic setting, and at least two chemotherapeutic regimens for the treatment of the metastatic disease [5]. It is a synthetic analogue of the marine natural product halichondrin B, isolated from the Japanese marine sponge *Halichondria okadai* [6, 7]. EM is a non-taxane microtubule dynamics inhibitor; indeed, its site and mechanism of action are different from other tubulin-targeting agents such as taxanes and vinca-alcaloïds [8]. EM inhibits microtubule polymerisation without affecting their depolymerisation, resulting in non-productive aggregates which induce an irreversible mitotic block at the G2-M phase, and leading to apoptosis [9]. The phase III pivotal open-label randomised study EMBRACE compared EM monotherapy *versus* treatment of physician’s choice (TPC) in patients with MBC. This trial showed the efficacy and tolerability of this molecule, leading to its approval by the US Food and Drug Administration (FDA) on November 15^th^, 2010. EMBRACE demonstrated a significant and clinically improvement in overall survival (OS) under EM treatment compared with the TPC in this setting. However, the vast majority of the patients included in this trial had previously been treated with capecitabine. Another phase III trial comparing EM *versus* capecitabine [10] in a population of 1102 patients previously treated with anthracyclines and taxanes did not show a statistically significant superiority of EM over capecitabine in terms of progression-free survival (PFS) and OS. However, considering the EMBRACE trial [11] and the toxicity profile in the Kaufman study, EM appears as a new therapeutic option in patients with metastatic or locally advanced breast cancer, and pre-treated with taxanes- and anthracyclines. Despite its extensive use in these patients, EM clinical efficacy and safety in the “real-world” patient population still need to be clearly evaluated.

This retrospective, international study aimed at evaluating EM activity and safety in a routine clinical setting, and at comparing our results with the published clinical data about EM.

## Methods

### Study design

Four centres participated in this retrospective clinical study, three centres in France (Institut régional du Cancer de Montpellier [ICM], Montpellier, Institut Claudius Regaud, Toulouse, and Centre Georges-François Leclerc, Dijon), and one centre in Switzerland (CHU Vaudois, Lausanne). An Access database was created to collect retrospective data using different panels: patients’ identification, tumour histology, previously delivered treatments and regimens, initial assessment, initial biology, EM chemotherapy-related data (delivered EM dose and cause of diminution, number of cycles administered, toxicities and side effects), efficacy and follow-up data. The tumour response was assessed every 3 cycles of treatment. Tumours were considered as ER and PR positive when > 10 % tumour cells were stained by immunohistochemistry (IHC). HER2 status was determined based on HER2 protein expression level by IHC. Tumours with HER2 scores of 0 and 1+ were considered as HER2 negative. In tumours with equivocal HER2 IHC test results (2+), gene amplification was evaluated using fluorescent (FISH) or chromogenic (CISH) in situ hybridization. Specimens with HER2 3+ scores were considered as HER2 positive. The Database locking and patients’ follow-up was scheduled for April 7^th^, 2014. This study was reviewed and approved by the respective Institutional Review Boards (Dijon, Toulouse and Montpellier Cancer Centres, and Lausanne CHUV, ID number ICM-URC-2014/73). Considering the retrospective, non-interventional nature of this study evaluating an approved drug, no consent was deemed necessary by the clinical research review board of Montpellier cancer centre (sponsor of the study).

### Patients

All patients affected by a metastatic or locally advanced breast cancer treated with EM between March 28^th^, 2011 and January 15^th^, 2014 in one of the participating centres, were included in our retrospective analysis. Patients with EM treatment initiated in other centres, and who received only one EM injection or cycle in a participating centre, were not considered suitable for this study due to the lack of data.

### Treatment

Eribulin mesylate was administrated intravenously over 2 to 5 minutes on days 1 and 8 of a 21-day chemotherapy cycle, according to the product guidelines. The EM treatment was generally administered at the standard dose of 1.4 mg/m^2^. It was reduced in case of hepatic or moderate renal impairments: a -20 % reduction of the recommended EM dose (1.1 mg/m^2^) was applied for patients with mild hepatic impairment (Child-Pugh A) or moderate renal impairment (creatinine clearance of 30–50 mL/min), and a −50 % reduction (0.7 mg/m^2^) was administered in patients with moderate hepatic impairment (Child-Pugh B). Clinical and radiological assessment of the tumour response was performed according to each centre standard of care, most of the time every 3 cycles of treatment (every 9 weeks). Clinical and biological assessment of toxicity was performed at each clinical visit, *i.e.* at day 1 and day 8 of each 21-day cycle. Primary and secondary granulocyte-colony stimulating factor (G-CSF) prophylaxis was delivered according to each centre’s practice.

### Efficacy assessment

Clinical and radiological efficacy assessment was performed every 3 cycles by a medical oncologist during the whole treatment period. Response and progression evaluations were performed using the RECIST version 1.1 criteria [12]. For each evaluation, treatment response was determined as such: complete response (CR), partial response (PR), stable disease (SD), progressive disease (PD), or not established (NE). Clinical proposal following assessment (continued treatment, dose reduction or treatment discontinuation) was recorded, together with the reasons of treatment discontinuation (toxicity, evolution, death or other).

### Safety assessment

Clinical and biological toxicities were retrospectively identified and graded according to the Common Terminology Criteria grid for Adverse Events (CTCAE) version 4.03 at each clinical visit, using the patients’ clinical charts. Interdose complications were recorded: treatment delay (duration and cause), treatment cancellation and reason, hospitalization (duration and cause), duration of antibiotic treatment, and need and number of Red Blood Cells (RBC) and/or platelet concentrates in case of transfusion.

### Statistical analysis

For the descriptive analysis, quantitative variables were presented for each group and for the overall population as mean, variance, standard deviation, minimum, maximum, and median. Quantitative criteria were compared using the Kruskal-Wallis test. Qualitative variables were presented as numbers and frequencies for each category of the variable. Qualitative criteria were compared using the chi2 or by the Fisher exact tests when the chi2 test was not applicable. The primary objective of the study was to assess overall survival until progression. Survival estimates were calculated using the Kaplan-Meier method. The time to progression was defined for each patient as the time from the first cycle until objective tumour progression (TTP does not include deaths). The secondary objective was overall survival (OS), defined as the time from the first cycle of treatment until death from any cause. For the clinicopathological features, univariate analyses to compare clinical benefit and no clinical benefit were performed using Pearson’s 2 or Fisher’s exact tests for categorical variables, and the two-sample Wilcoxon test for all continuous variables. Categorical covariates analysis were ECOG Performance Status (0–1 *vs*. 2–3), hormone receptors (HR- *vs*. HR+), and age (≤50 *vs*. > 50 years), HER2+ over-expression (no/yes), number of prior chemotherapy (≤4 *vs*. > 4 lines) and different metastasis localization (visceral metastases, liver, bone, lymph nodes, lung, brain, serous or skin metastases), and response under taxane chemotherapy (CR/PR/SD *vs*. PD/NE). Differences were considered statistically significant when *p* < 0.05. Significant factors in the univariate analyses were included in a multivariate logistic regression analysis to identify independent predictors of the clinical benefit.

For the multivariate analysis using the Cox’s proportional hazard model to define independent prognostic factors for PFS, the variables included in the logistic regression were used. The hazard ratio and the 95 % confidence interval (CI) were also estimated.

Different bases transfers were made through STAT-TRANSFER version 9 and data were analysed using the STATA® version 13.0 software.

## Results

### Patients

Two hundred and fifty-eight patients were included in our study. Main clinic pathological characteristics of the population are presented in Table 1. Median age at breast cancer diagnosis and at EM initiation was 50 (18–80) and 59 years old (22–85), respectively. Patients’ ECOG performance status at EM initiation was 0 or 1 in 79.9 % of the cases. Biological subtypes were classical for this population, as 73.3 % of the tumours were Hormone Receptor (HR)-positive, 10.2 % were HER2-positive, and 22.5 % were triple negative (TN). Disease extension was typical of late stage tumours: 86.4 % of the patients presented with visceral metastases, mainly in the liver (67.4 %). Patients with HR-positive tumours had previously been treated with at least one hormonal therapy line for metastatic disease in 97.3 % of the cases. Patients were heavily pretreated with chemotherapy (median number of previous metastatic regimens was 4 [1–9]) in the metastatic setting. All patients had previously received anthracycline- and/or taxane-based treatments (94.6 % of the patients had previously received anthracycline and 98.1 % taxane regimen in neoadjuvant/adjuvant and/or metastatic chemotherapy settings). The majority (82.1 %) of patients pretreated with taxanes showed primary taxane sensitivity as defined by CR, PR or SD > 6 months under taxane treatment in metastatic setting. Twenty-five of the 26 patients presenting HER2-positive tumours received trastuzumab concomitantly with EM administration.

**Table 1:** Main patient and tumour characteristics (*n* = 258)

Characteristics	
Median age at diagnosis; years (range)	50 (18–80)
Histological type of primary tumour; n (%)	
Ductal	228 (88.4)
Lobular	21 (8.1)
Other subtypes	9 (3.5)
Tumour subgroup; n (%)	
ER-positive	184 (71.32)
PR-positive	122 (47.29)
HR-positive	189 (73.3)
HER2-positive	26 (10.2)
Triple negative	58 (22.5)
ECOG performance status (%)	
0	73 (28.3)
1	133 (51.6)
2	46 (17.8)
3	6 (2.3)
Previous chemotherapy for (neo) adjuvant/advanced disease	
Anthracyclines; n (%)	244 (94.6)
Taxanes; n (%)	253 (98.1)
Capecitabine; n (%)	234 (90.7)
Median prior lines of chemotherapy in the metastatic setting (range)	4 (1–9)
Best response under previous taxane therapy; n (%)	
Complete response	20 (7.8)
Partial response	112 (43.8)
Stable disease	78 (30.5)
Progressive disease	33 (12.9)
Not evaluable	13 (5.1)
Missing	2
Hormonal therapy	
Prior hormonal therapy (HR+ tumours; n [%])	184 (97.3)
Metastatic sites; n (%)	
Visceral metastases	223 (86.4)
Liver	174 (67.4)
Node	118 (45.7)
Lung	98 (38.0)
Brain	41 (15.9)
Serous	55 (21.3)
Skin	31 (12.0)
Bone	176 (68.2)
Other sites	26 (10.1)

### Eribulin mesylate chemotherapy

The median number of delivered EM cycles was 5 (1–19) (Table 2). EM was first administered at the full recommended dose (1.4 mg/m^2^) in 79.1 % of patients. Causes of dose reductions at treatment initiation were due to liver function impairment in 57.7 %, persistent haematological toxicity in 11.5 % and other causes in 13.5 %; cause of dose reduction was not indicated in 17.3 % of cases. Dose reduction under treatment was due to haematological toxicity (all grades) in 20.2 % of cases, liver toxicity in 13.2 % of cases and to other causes (14.3 %). The relative dose intensity was 0.917 (0.165–1.22). Two hundred and thirty-six (91 %) patients had discontinued EM treatment at the date of analysis. The most common cause of treatment discontinuation was disease progression (75 %), followed by toxicity (13.1 %), and patient’s death (8.1 %); also, 7.2 % of the patients discontinued EM treatment due to other causes such as patient’s or medical decision.

**Table 2:** Efficacy and drug exposure

Objective response rate; n (%)	65 (25.2)
Median duration of objective response, months	4.4 (95 %CI: 3.4–4.8)
Best overall response	
Complete response; n (%)	1 (0.4)
Partial response; n (%)	64 (24.8)
Stable disease (>6 months); n (%)	28 (10.9)
Clinical benefit rate, % (95 %CI)	36.1 (30.2–42.2)
Progressive disease; n (%)	157 (60.9)
Not established; n (%)	8 (3.1)
Median time to progression, months ( 95 %CI)	4 (3.3–4.3)
Overall survival, months (95 %CI)	11.2 (9.3–12.1)
One- year overall survival rate,% (95 %CI)	45.5 % (38.3–52.4)
18-month overall survival rate, % (95 %CI)	23.1 % (16.3–30.6)
2-year overall survival rate, % (95 %CI)	8.5 % (2.2–20.3)
Drug exposure	
Median EM cycles delivered; n (range)	5 (1–19)
Dose reductions at initiation (%)	20.9
Relative dose intensity (range)	0.92 (0.17–1.22)

### Efficacy

**Fig. 1:**
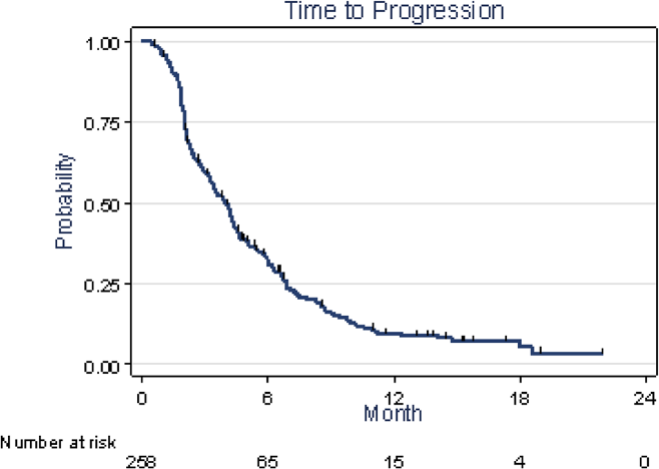
Time to progression

**Fig. 2:**
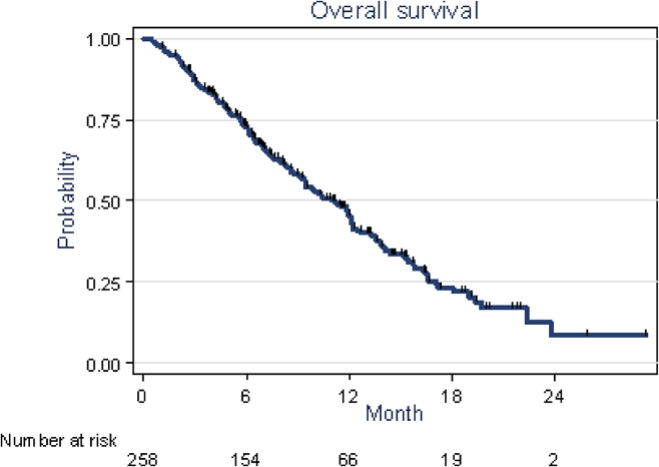
Overall survival

At the time of analysis, after a median follow-up of 13.9 months (95 %CI: 11.66–15.60), 42.3 % of the patients were still alive. Death was mainly related to disease progression (PD, 95.1 %). Toxic death was reported in 2 cases (1.4 %) and was related to intercurrent diseases in 5 cases (3.5 %). Concerning treatment and progression, 38 % of the patients were still under treatment and 55.5 % reported disease progression. One patient presented a complete response to treatment and 64 patients (24.8 %) showed a partial response (PR). Thus, the objective response rate was 25.2 % (95 %CI: 20–31). The median duration of objective response was 4.36 months (95 %CI: 3.38–4.82). Moreover, a disease stable for at least 6 months (SD) was observed in 28 patients (10.9 %) and 157 (60.8 %) showed PD. The tumour response could not be evaluated in 8 patients (3.1 %) due to premature discontinuation of the treatment related to toxicity. The clinical benefit rate, defined as CR rate, PR rate and SD during at least 6 months, was 36.1 % (Table 2). Median TTP and OS were 3.97 (95 %CI: 3.25–4.3) and 11.2 (95 %CI: 9.3–12.1) months, respectively (Figs. 1 and 2 respectively). Twelve-, 18- and 24-month overall survival rates were 45.5 % (95 %CI: 38.34–52.36), 23.13 % (95 %CI: 16.34–30.63) and 8.5 % (95 %CI: 2.21–20.30), respectively (Fig. 2).

### Safety

The EM treatment was globally well tolerated. Table 3 summarizes the main grade 3–4 toxicities reported in our population. The most commonly reported toxicities were asthenia (60.9 %), peripheral neuropathy (43 %), neutropenia (38.4 %), alopecia (19.4 %), nausea (10.5 %) and thrombocytopenia (10.5 %). Major toxicities were of grade 3 (39.5 %) and grade 4 (17 %), and were as follows: neutropenia (20.9 %), peripheral neuropathy (3.9 %), anaemia (1.6 %), liver dysfunction (0.8 %), and thrombocytopenia (0.4 %). Thirteen patients (5 %) developed febrile neutropenia.

**Table 3:** Main toxicity in 258 patients according to cTcAE version 4.03

Grade 3–4 toxicities; n (%)	
Anemia	4 (1.6)
Neutropenia	54 (20.9)
Thrombocytopenia	1 (0.4)
Liver dysfunction	2 (0.8)
Peripheral neuropathy	10 (3.9)
Febrile neutropenia	13 (5.0)

A treatment delay was reported in 69 patients (26.7 %). The average number of days of report was 14.9 (SD σ = 16.8) with a median number of 8 days (3–130 days). The delay was mostly due to treatment toxicities (60.9 %). Primary G-CSF prophylaxis was used in 15.1 % of the patients and as secondary prophylaxis in 12.4 % of the cases. Antibiotics during EM treatment were used for 17 % of patients, and red blood cells and platelet concentrates transfusions in 6.2 and 0.4 %, respectively. Hospitalisation was required for 17.8 % of the patients due to cancer-related complications in 52.2 % of cases, treatment toxicity in 34.8 % and other causes in 37.0 %.

### Predictive and prognostic factors

Response and survival rates were evaluated in regards of the usual prognostic factors to identify relevant prognostic and predictive factors affecting this population. Using a logistic regression analysis, HER2 positivity was significantly associated with higher CBR (HR = 0.38, *p* = 0.02); the presence of serous metastases was of borderline significance (HR = 0.55, *p* = 0.052), while a TN status and the presence of lung metastases were significantly associated with lower CBR rates (HR = 2.04, *p* = 0.044 and HR = 2.16 and *p* = 0.006, respectively). The achievement of a clinical benefit under a previous taxane therapy was of borderline significance (*p* = 0.054). In multivariate analysis, HER2 positivity (OR = 0.26; 95 %CI 0.10–0.63) was an independent favourable predictive factor, while the presence of lung metastases (OR = 2.49; 95 %CI 1.43–4.61) and the inability to achieve a clinical benefit under a previous taxane therapy (OR = 2.36; 95 %CI 1.11–5.03) were independently associated with a lower CBR.

Using the Cox regression model, a difference was observed regarding TTP: in univariate analysis, HR positivity and the presence of serous metastases were significantly associated with a longer TTP, while a TN status, the inability to achieve a clinical benefit under a previous taxane therapy and the presence of lung metastases were significantly associated with a shorter TTP. In multivariate analysis, HR positivity (HR = 0.68; 95 %CI 0.51–0.92), the presence of lung metastases (HR = 1.53; 95 %CI 1.16–2.02) and the inability to achieve a clinical benefit under a previous taxane therapy (HR = 1.50; 95 %CI 1.07–2.11) were the only 3 independent prognostic factors of this population.

Focusing on the TN subgroup, the overall response rate (ORR) was significantly higher in the TN population (respectively 26.9 % *vs*. 22.8 %, *p* = 0.002) compared with non-TN breast cancers. However, the OS and the TTP were significantly lower (respectively, 8.3 months *versus* 11.9 months, *p* = 0.049, HR [95 % CI] = 1.46 [1.01–2.12]; 2.1 months *versus* 4.3 months, *p* = 0.0004, HR [95 % CI] = 1.80 [1.32–2.45]) in the TN population.

## Discussion

To our knowledge, our study is the largest international multicentre retrospective study of EM use in heavily pretreated breast cancer patients. Our results confirm the EM efficacy and safety in the daily care treatment of heavily pretreated MBC patients, with a population exposed to a median of four prior lines of chemotherapy. The results of our study are comparable to those of the pivotal EMBRACE phase III trial [13]. The two populations appeared relatively similar regarding the median age (59 *versus* 55 years old) and the metastatic sites distribution (Table 4). The rate of HER2+ tumours was discretely lower in our population (10.2 % *versus* 16 %) while the rate of TN tumours was slightly higher (22.5 % *versus* 18 %) in the EMBRACE trial. TN tumours seemed to respond better to the treatment, as we observed an increased ORR in the TN subpopulation compared with other biological subgroups (*p* = 0.002). However OS and TTP were significantly lower. Moreover, HER2 positivity appeared as a predictive factor, with 57.7 and 33.9 % CBR for HER2+ and HER2- tumours, respectively. These results, which strikingly differ from the previous results from randomized trials, in which none of the patients received concomitant trastuzumab, may be due to the nearly systematic use of trastuzumab in association with EM in our population. Indeed, in these studies, HER2 had no impact on EM efficacy [11, 13]. A recent phase II study evaluating the safety and efficacy of the EM – trastuzumab association in a first-line metastatic setting demonstrated a good safety profile and an interesting 71.2 % ORR [14]. The efficacy of this treatment combination could explain our results and warrant further evaluation of this association. Nevertheless, other studies will be needed to thoroughly assess these differences in a clinical behaviour. The proportion of patients with HR+ tumour was higher in our study compared with the EMBRACE trial (73.3 % *versus* 64 %). Almost all these patients had previously been treated with hormone therapy (97.3 %). Considering the independent prognostic value of the HR+ status in our population, this fact could explain the relatively similar OS results obtained compared with the EMBRACE trial patients, this favourable prognostic factor counter-balancing the classical lower results of retrospective studies due to the lack of selection in a daily-care setting population. Also, some recent preclinical data showed that EM may have some additive antitumoral effect on estrogen-stimulated ER-positive breast cancers [15].

**Table 4:** Comparative evaluation between the EMBRACE and the ERIBEX studies

	EMBRACE	ERIBEX
Population		
Patients (n)	503 (EM)	258
Age (years)	55	59
Triple-negative tumour (%)	18	22.5
HER2+ tumour (%)	16	10.2
Prior hormonal therapy (%)	85	76.7
Prior anthracycline or taxane treatment (%)	99	100
Prior capecitabine treatment (%)	73	90.7
Median prior metastatic chemotherapies; n (range)	4 (1–7)	4 (1–9)
Metastatic sites		
Bone (%)	61	68.2
Liver (%)	60	67.4
Node (%)	44	45.7
Lung (%)	38	38
Chemotherapy		
Median number of EM cycles (range)	5 (1–23)	5 (1–19)
Efficacy		
Median overall survival (month, 95 %CI)	13.1	11.2 (9.3–12.12
Median progression-free survival (month, 95 %CI)	3.7	3.8 (3.2–4.2)
Overall response rate (%)	12	26
Clinical benefit rate	23	36
Safety		
Side effects (grade 1–4, %)	99	94.2
Side effects most commonly encountered		
Asthenia (%)	54	60.9
Neutropenia (%)	52	38.4
Peripheral Neuropathies (%)	35	43
Nausea (%)	35	10.5
Alopecia (%)	45	19.4
Grade 3 toxicity (%)	36.2	39.5
Grade 4 toxicity (%)	27.2	17
Grade 3–4 neutropenia	21 %/24 %	20.9 % grade 3–4
Grade 3-/4 peripheral neuropathy	8 %/<1 %	3.9 % grade 3–4
Treatment discontinuation due to toxicity (%)	13	13.1

Our population was heavily pretreated, as EM was initiated as a fifth-line of treatment, a line comparable to that of the EMBRACE study. The anthracyclines and/or taxanes pretreatment rate was comparable to the one in the phase III study, reflecting the high adherence of the participating institutions to breast cancer care recommendations [3]. In addition, for the majority of patients pretreated with taxanes, the best response rate (CR or PR) observed in our study was 51.6 %, with 82.1 % CBR under taxane therapy, defining a population of patients showing good primary taxanes sensitivity. As taxanes and EM share the same cellular site of action, this parameter appears to be a logical predictive variable of EM sensitivity. This assumption was verified in our series, as patients without evidence of primary taxane sensitivity in a previous line appeared to have statistically lower CBR and TTP under EM in multivariate analysis. However, this result needs to be confirmed in a separate independent population. Also, the capecitabine pretreatment rate was greater in our study (90.7 %) compared with the rate reported in the EMBRACE study (73 %).

To date, no clinical study demonstrated the superiority of EM compared to capecitabine. The phase III study conducted by Kaufman *et al*. comparing EM to capecitabine did not show a statistically significant superiority of EM over capecitabine in terms of OS and PFS, in a population of patients who had received up to two previous lines of chemotherapy for metastatic disease (including anthracycline and taxane) [10, 11]. In the EMBRACE study, the EM group of patients was predominantly capecitabine-pretreated, which may explain the choice of capecitabine use before EM in routine clinical practice. The convenience provided by an oral administration of capecitabine may also explain its frequent use prior to EM treatment.

Considering the efficacy issue, our results also appeared globally similar to the results of the pivotal phase III EMBRACE trial, with an OS of 11.2 and 13.1 months respectively, and a PFS of 3.8 and 3.7 months, respectively (Table 4). Interestingly, the ORR and CBR in our retrospective, unselected study were relatively higher compared with the EMBRACE study results (25.2 % *versus* 12 % and 36.1 % *versus* 23 % respectively). Even if no definitive conclusion can be issued from such an indirect comparison, these results are interesting as clinical practice results frequently appear less favourable than pivotal trials results. In our study, EM treatment was most of the time initiated at full dose (79.1 %). Major reported cause of dose reduction was liver dysfunction. At the same time, percentage of prescription of primary or secondary G-CSF prophylaxis was relatively low (15.1 and 12.4 % respectively). These results could possibly be explained by the choice of considering lower doses of EM instead of using a G-CSF prophylaxis in 2 weeks over 3 schedules.

Treatment discontinuations due to EM toxicity were relatively rare in our experience. Similarly, only few hospitalizations and transfusions were required, demonstrating a good safety profile for the treatment. Alopecia was relatively rare in our series (19.4 % *vs*. 45 % in the EMBRACE study). This low alopecia frequency could be linked to preventive measures routinely used in our centres, such as the use of a cooling helmet during treatment. Another possible explanation is the more frequent use of capecitabine in the previous therapeutic line, leaving a greater proportion of patients without grade 2 alopecia at initiation of the EM treatment. The overall safety profile of our series appears otherwise comparable to the results of the EMBRACE study (Table 4).

Our study shows the limitations commonly observed in retrospective studies (many items are not defined from a predefined study protocol, the study is lacking a central immunohistochemistry lecture…). However, the greatest strength of our study is that it represents a large data from a cohort of patients not included in clinical trials, thus better reflecting the real-life patient population in terms of comorbidities and disease extension. Table 5 summarizes the main results of the presently published data on EM in metastatic breast cancer, including the pilot phase III EMBRACE trial [13], the study reported by Kaufman *et al*. comparing EM with capecitabine [10], together with recent retrospective studies performed in daily care conditions published in 2014, as well as our study data, giving consistent results to ensure the use of EM in the heavily pretreated MBC setting, in terms of clinical efficacy and with a favourable efficacy/safety ratio.

**Table 5:** Summary of data published from phase III-IV trials on eribulin mesylate in metastatic breast cancer

Study (years)	Phase	Treatment	Patients (n)	OS (months)	PFS (months)	DOR (months)	OR (%)	Ref.
Cortes *et al*. (2011) [13]	III	Eribulin mesylate *vs* TPC	508 *vs* 254	13.1	3.7	3.9	13	[6]
Kaufman *et al*. (2012) [10]	III	Eribulin mesylate *vs* Capecitabine	554 *vs* 548	15.9	4.2	4.1	11	[10]
Ramaswami *et al*. (2014) [16]	IV	Eribulin mesylate	25	5.89	4.08	-	16	[16]
Poletti *et al*. (2014) [17]	IV	Eribulin mesylate	27	8	-	2.5	9	[17]
Gamucci *et al*. (2014) [18]	IV	Eribulin mesylate	133	14.3	4.4	5.2	21.1	[18]
Rasmussen *et al*. (2014) [19]	IV	Eribulin mesylate	44	-	-	-	12.5	[19]
Present study	IV	Eribulin mesylate	258	11.2	3.8	4.4	26	

*DOR* Duration Of Response, *OR* Objective Response, *OS* Overall Survival, *PFS* Progression-free survival, *TPC* Treatment of physician’s choice

## Conclusion

The evaluation of new drugs in current practice, outside clinical trials, remains a key element of the evaluation of their efficacy and safety after large pivotal studies. The results showed by current practice could reveal to be different from the results obtained during the clinical trials step, usually because of the heterogeneity of the analysed population treated in routine conditions and under less stringent selection than that of the trials. In our study, EM appears to be an effective new-line treatment in heavily pretreated MBC patients, with a favourable efficacy/safety ratio. These encouraging results compare favourably with those obtained in the pivotal phase III EMBRACE study and with more recent retrospective studies. They comfort the use of EM in the heavily pretreated MBC setting. Tumour biology, primary taxanes sensitivity and metastatic sites could represent useful predictive and prognostic factors in this population.

## Abbreviations

CISH: Chromogenic in situ hybridization
CBR: Clinical benefit rate
CR: Complete response
CTCAE: Common Terminology Criteria grid for Adverse Events
EM: Eribulin mesylate
FISH: Fluorescent in situ hybridization
G-CSF: Granulocyte-colony stimulating factor
HR: Hormone Receptor
IHC: Immunohistochemistry
MBC: Metastatic breast cancer
NE: Not evaluable
OS: Overall survival
PFS: Progression-free survival
RBC: Red blood cells
TN: Triple negative
TPC: Treatment of physician’s choice
TTP: Time to progression
PD: Progressive disease
PR: Partial response
SD: Stable disease
